# Microfluidic Electrochemical Glucose Biosensor with In Situ Enzyme Immobilization

**DOI:** 10.3390/bios13030364

**Published:** 2023-03-09

**Authors:** Nina Lokar, Borut Pečar, Matej Možek, Danilo Vrtačnik

**Affiliations:** Laboratory of Microsensor Structures and Electronics, Faculty of Electrical Engineering, University of Ljubljana, Tržaška 25, 1000 Ljubljana, Slovenia

**Keywords:** microfluidic glucose biosensor, electrochemical biosensor, thin film electrode, enzyme in situ immobilization, chronoamperometry, cyclic voltammetry

## Abstract

The development and characterization of a microfluidic electrochemical glucose biosensor are presented herein. The transducer part is based on thin-film metal electrodes on a glass substrate. The biological recognition element of the biosensor is the pyrroloquinoline quinone–glucose dehydrogenase (PQQ-GdhB) enzyme, selectively in situ immobilized via microcontact printing of a mixed self-assembling monolayer (SAM) on a gold working electrode, while the microfluidic part of the device comprises microchannel and microfluidic connections formed in a polydimethylsiloxane (PDMS) elastomer. The electrode properties throughout all steps of biosensor construction and the biosensor response to glucose concentration and analyte flow rate were characterized by cyclic voltammetry and chronoamperometry. A measurement range of up to 10 mM in glucose concentration with a linear range up to 200 μM was determined. A detection limit of 30 µM in glucose concentration was obtained. Respective biosensor sensitivities of 0.79 nA/µM/mm^2^ and 0.61 nA/µM/mm^2^ were estimated with and without a flow at 20 µL/min. The developed approach of in situ enzyme immobilization can find a wide number of applications in the development of microfluidic biosensors, offering a path towards continuous and time-independent detection.

## 1. Introduction

The synergy of biosensors, electrochemistry and microfluidics has been signaling a new era in science and technology in recent years [1]. One example of this overlap is an electrochemical biosensor on a chip [2]. Compared to other analytical methods, the advantages of biosensors are on-site detection, real-time detection and ease of use. Biosensors are also useful for preliminary analyses before more complex and expensive analyses are performed. They also enable simultaneous detection of multiple analytes [3]. Recently, portable smartphone-based readers have been developed to monitor analytes in biofluids [4,5,6,7,8]. These also have many applications, with special emphasis on wearable biosensors [9,10]. Due to the diversity of biosensors, which comprises a variety of combinations of bio-recognition elements and transducer elements, a wide range of biosensor applications is possible [1]. Optical biosensors are among the most common types of biosensors [11,12,13]. One example of these is based on localized surface plasmon resonance phenomenon for the real-time detection of biomolecules [14,15,16,17]. In addition, reliable, practical and relatively inexpensive electrochemical biosensors have been developed [3,18]. In terms of the biological recognition element, electrochemical biosensors are of particular interest for the integration of enzymes that are highly selective for specific analytes [1]. Currently used commercial biosensors are still large in size, slow in operation, expensive and labor-intensive; thus, it is difficult to automate, integrate and miniaturize existing conventional devices for multiple applications. There are numerous strategies to design and fabricate miniaturized biosensors. One of them is microfluidics, which has been widely explored [3,19,20,21,22]. Integrating a biosensor into a microfluidic chip brings miniaturization with extremely low sample consumption and continuous measurement of the analyte. The microfluidic approach also enables self-calibration with an internal analyte standard and the development of automated analyte control with a feedback loop based on real-time measurements of the analyte in the bioprocess. There is also the possibility of automatic selection or exchange between sensory elements [3,18].

Microfluidic biosensors are used to improve quality of life in various fields such as environmental monitoring [23,24], medicine [25,26], the food industry [27] and security and defense and drug discovery [19]. Some examples of microfluidic electrochemical enzyme biosensors are those for the measurement of cholesterol [28,29], thyroid drugs [30] and adenosine-5’-triphosphate [31].

In this paper, we present the development and characterization of a microfluidic electrochemical pyrroloquinoline quinone–glucose dehydrogenase (PQQ-GdhB) biosensor, which has the potential for continuous glucose monitoring in biopharmaceutical process control. Compared to other glucose-oxidizing enzymes, PQQ-GdhB has a long list of advantageous properties. PQQ-GdhB displays one of the highest catalytic efficiencies while remaining highly selective for glucose. In contrast to fungal glucose oxidase, the reaction mechanism with PQQ-GdhB does not depend on oxygen levels in the system and does not generate reactive byproducts such as H_2_O_2_. The PQQ cofactor is tightly bound with the GdhB and no additional soluble cofactors (i.e., NAD(P)+, FAD+) are needed for the reaction [32]. Successful electron transfer from PQQ-GdhB has been demonstrated to both solid-phase [33,34,35] and soluble electron acceptors [36]. The latter is also true when PQQ-GdhB is immobilized, which allows independent measurements of certain properties of electrochemical biosensors. In our work, PQQ-GdhB was immobilized on a gold electrode using a mixed self-assembling monolayer (SAM) of 6-mercaptohexanol and 11-mercaptoundecanoic acid (6-MCH+11-MUA), crosslinked by 1-ethyl-3-(3-dimethylaminopropyl)carbodiimide and N-hydroxysulfosuccinimide (EDC+S-NHS), similar to the approach presented in [37]. The main challenge in developing a microfluidic system for a biosensor was to integrate the biological components with the fabricated transducers and microfluidic components. To immobilize the enzyme only on the working thin-film electrode, several integration approaches can be found in the literature, such as laminar co-flow with a carefully selected electrode position in the microchannel [38], applying a potential to the electrode that electrophoretically attracts the biological component to the electrode [39], and the electro-click chemistry method in a completely packaged freestanding channel [40]. Herein, we developed a method for selective microcontact printing [41] of mixed SAM onto a thin-film gold working electrode (WE). The key element of microcontact printing is a polymeric stamp with a relief pattern. This stamp is “inked” and put in contact with the WE surface. The ink is transferred from the stamp to the substrate only in the area of contact. After that, a microchannel was formed on the electrode chip, so both the activation of 11-MUA and the immobilization of the enzyme were performed with a controlled flow of substances through the microchannel. In this way the fabricated biosensor chip can be prepared in advance, without the enzyme, which can be immobilized just in time, i.e., just before using the sensor, which keeps the sensing area dry and inactive until the test begins. This approach reduces the burden during a microfluidic biosensor fabrication, keeping the required reagents stored outside of the detection structure in suitable wet conditions. In addition, our approach using the microcontact printing method reduced the time for mixed SAM deposition from several hours [37] or even a day to a few minutes compared to the conventional deposition approach by immersing the electrodes in the solution. Finally, miniaturization of the biosensor was implemented and proved to be advantageous in terms of sensor response magnitude and analyte consumption.

## 2. Materials and Methods

### 2.1. Materials and Instruments

For the biological part of biosensor construction, the following chemicals were used: 97% purity 7 mM 6-MCH (Merck, Darmstadt, Germany), 95% purity 5 mM 11-MUA (Merck, Darmstadt, Germany), 200 mM EDC (Thermo Fisher Scientific, Waltham, MA, USA) and 50 mM S-NHS (Merck, Darmstadt, Germany). The enzyme solution was a mixture of 8 μL 4.2 μg/mL PQQ-GdhB from Acinetobacter calcoaceticus (Novartis Technical Operations, Mengeš, Slovenia) [36], 20 μL 95% 5 μM pyrroloquinoline quinone (PQQ) (Merck, Darmstadt, Germany), 40 μL 97% 10 mM magnesium chloride (MgCl_2_) (Merck, Darmstadt, Germany) and 732 μL phosphate-buffered saline (PBS) with pH 7.2 (Gibco, Thermo Fisher Scientific, Waltham, MA, USA). The optimal concentration of the enzyme was chosen based on preliminary immobilization tests. For this purpose, various dilutions of purified GdhB were used for immobilization onto gold plated inserts, which were placed into standard 96 microtiter plates. Mixed SAM was used for immobilization (see Section 2.2); however, immersion rather than printing was used. To compare initial velocities, the spectrofluorometric method according to reference [36] was modified so that the final electron acceptor was resazurin instead of 2,6-dichlorophenolindophenol. The fluorescence of the reduced resazurin was measured at the wavelengths 530 (excitation) and 590 (emission) nm.

For polydimethylsiloxane (PDMS) microchannel fabrication, a two-part kit Sylgard 184 (Dow Corning Corporation, Midland, MI, USA) was applied in the standard mixing mass ratio of 10 parts a pre-polymer (base) and 1 part a crosslinker (curing agent). Biosensor measurements were performed using the following chemicals: 2 mM ferri/ferro-cyanide ([Fe(CN)_6_]^3−/4−^) (Merck, Darmstadt, Germany) in 0.1 M potassium chloride (KCl) (Fluka, Bornem, Belgium) and 0.1 mM ferrocenemethanol redox couple (FcMeOH/FcMeOH^+^) (Merck, Darmstadt, Germany) in 0.1 M PBS with pH 7.2 (Gibco, USA), D-(+)-glucose (Merck, Darmstadt, Germany) dissolved in [Fe(CN)_6_]^3−/4−^ in KCl solution or FcMeOH in PBS solution. For basic cleaning and rinsing, isopropyl alcohol (Microchemicals, Ulm, Germany) and high-purity deionized water (in situ prepared) were used.

Electrochemical measurements were carried out using a potentiostat, EmStat3+ Blue, equipped with the PSTrace 5.9 software (PalmSens BV, GA Houten, The Netherlands). In some experiments, miniature Ag/AgCl with a 3 M KCl reference electrode, model ET073 (eDAQ Pty Ltd., Denistone East, NSW, Australia), was applied. For fabrication of PDMS microchannels, a Si mold was made by deep reactive ion etching using a Bosch technique (System 100 ICP 180, Oxford Instruments Plasma Technology, Bristol, UK). The plasma surface treatment system Atto (Diener electronic GmBh, Plasma Surface Technology, Nagold, Germany) was used for surface modification.

### 2.2. Design and Fabrication of Microfluidic Electrochemical PQQ-GdhB Biosensor

**Figure 1:**
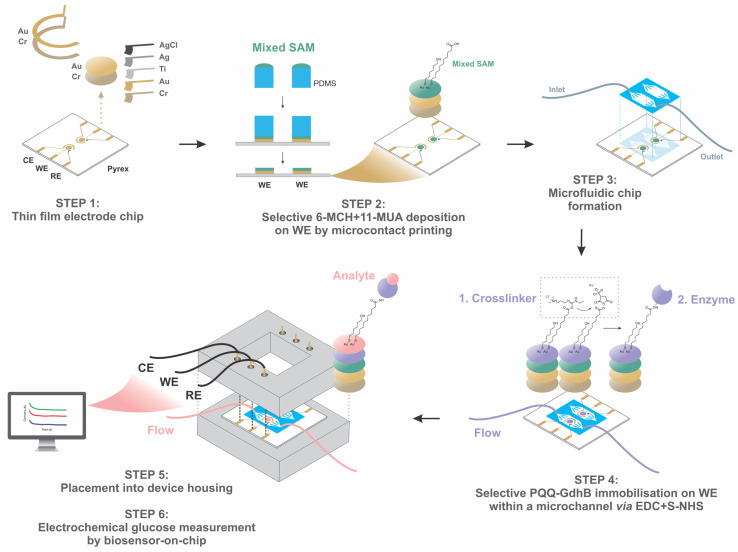
Construction of electrochemical PQQ-GdhB biosensor on a chip for glucose measurement. Step 1: Thin-film working and counter Au electrodes with Ag/AgCl reference electrode; Step 2: Selective adsorption of thiols’ SAMs, Au-MCH and Au-MUA, performed by microcontact printing; Step 3: Electrode chip integration with PDMS microchannel chip; Step 4: Esterification of the COOH functionalities by EDC and S-NHS crosslinker, resulting in activated Au-MUA and selectively covalent binding of the enzyme (Au-MUA-enzyme); Step 5: Biosensor chip placement into a custom-made housing; Step 6: Electrochemical detection with a potentiostat connected to a PC.

Figure 1 shows the sequence of the main process steps in the development of a microfluidic electrochemical enzyme biosensor for the detection of glucose. These steps are further described below.

*Step 1: Thin-film electrode chip*. The electrode chip was designed to provide two triplets of thin-film electrodes to enable parallel run tests. To fabricate them, Cr/Au layers (30/120 nm) were sputtered onto a precleaned Pyrex substrate. WE, counter (CE) and reference (RE) Au electrodes, Au conductive lines and Au connection pads were defined by photolithography and subsequent selective wet etchings of Cr/Au layers. Similarly, RE made from Ti/Ag thin films (40/1200 nm) was patterned next to WE and CE on the Cr/Au layer. The Ag layer was then partially transformed to Ag/AgCl electrode by the chemical oxidation reaction with FeCl_3_, according to Equation (1) [42]:

(1) $$\mathrm{Ag}+{\mathrm{FeCl}}_{3}\;⇄\;\mathrm{AgCl}+{\mathrm{FeCl}}_{2}$$

To avoid the contamination of neighboring WE and CE Au electrodes, a selective drop-casting method was utilized to apply FeCl_3_ for 1 min on the Ag electrode to confine the reaction exclusively to the RE area. The resulting Ag/AgCl thin-film electrode is considered a quasi-Ag/AgCl reference electrode [18,43].

*Step 2: Selectively mixed SAM deposition on WE*. The electrode chip was thoroughly cleaned, starting with wet cleaning in isopropyl alcohol, followed by ultrasonic cleaning in a 3:2 volume ratio isopropyl alcohol–deionized water mixture, and finally cleaning with oxygen plasma for 30 s at a working pressure of 1.0 mbar and an RF power of 50 W. After cleaning, the WE was modified by a mixed solution of 6-MCH and 11-MUA in ethanol to form, via a strong Au-thiolate bond, an oriented monolayer with its carboxylic group located at the monolayer–air or monolayer–liquid interface. 6-MCH was included to ensure that there is enough space for the enzyme to bind to 11-MUA. This mixed SAM selective deposition was performed by microcontact printing. Separately fabricated stamp chips from PDMS with the same shape and size as the WEs were firstly cleaned with oxygen plasma to form a hydrophilic surface, on which mixed SAM was then deposited by a pipette drop. After a few minutes, excess mixed SAM was removed by gently drying with N_2_. The stamp with only a thin layer of mixed SAM was precisely aligned on the WEs of the electrode chip for 20 s. Afterward, excess mixed SAM was removed by thoroughly rinsing the entire electrode chip with ethanol, followed by ultrapure water and drying with N_2_.

*Step 3: Microfluidic chip formation*. The PDMS microchannel was fabricated by soft lithography using a replica molding technique. The mold was fabricated by deep reactive ion etching of a patterned Si wafer. The PDMS replica formed was then exposed to an oxygen plasma with 0.8 mbar pressure and 30 W power for 20 s. The activated PDMS surface was pressed to the glass electrode chip and was irreversibly chemically bonded. The microfluidic connection to the chip was made with the polymer Tygon tubes, which were connected to the PDMS microchannel via stainless steel tube connectors.

*Step 4: Selective enzyme immobilization on WE*. First, 11-MUA was activated by a crosslinker, which involved stepwise formation and replacement of terminal EDC and S-NHS in sequence to form an S-NHS ester. Secondly, the active S-NHS ester was replaced by the primary amines of PQQ-GdhB, thus immobilizing the enzyme via the amide bond. This was performed in situ, i.e., by continuous flow of the crosslinker and the enzyme within the microchannel at a flow rate of 2 µL/min with exposure time of 15 and 120 min, respectively. Finally, this resulted in selective enzyme immobilization on mixed SAM-coated WE.

*Step 5: Placement of the electrode chip with microchannel into the housing*. The biosensor chip was installed in a custom-designed polyethylene housing that contained spring gold-coated pin connectors to ensure a reliable electrical connection between the electrodes and potentiostat. This configuration enables two sets of measurements to be performed simultaneously or in a sequence. To avoid mechanical damage of the glass, a custom-made gasket from PDMS was provided. Precise alignment of the electrode chip in the housing was achieved with a groove in the base and three insertion metal pins on the cover part of the housing (not shown).

### 2.3. Experimental Setup

**Figure 2:**
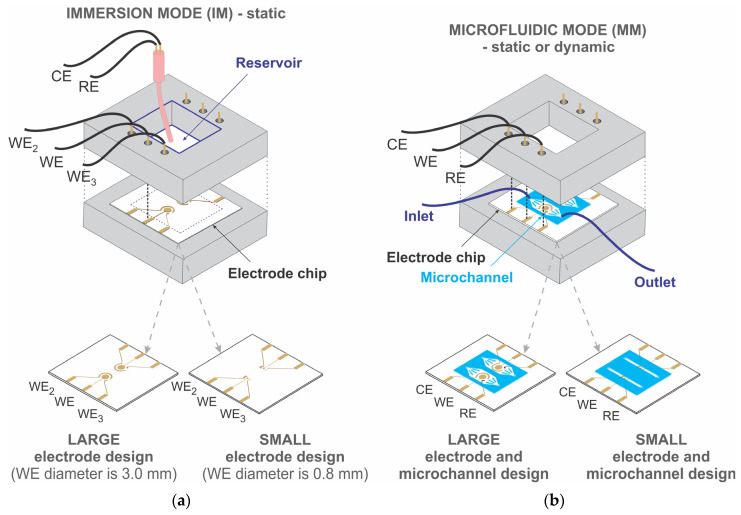
Two approaches of biosensor construction: (**a**) immersion mode (IM) biosensor—thin films WE, WE_2_, WE_3_ and externally immersed CE and RE.; (**b**) microfluidic mode (MM) biosensor—thin films WE, CE and RE, which are integrated into the microchannel. Both modes include large and small electrode and microchannel designs.

Two measurement modes were used to characterize the developed biosensors, namely the immersion mode (IM), which allows static measurements, and the microfluidic mode (MM), which allows either static measurements or measurements during continuous flow of the solution. Each mode was tested with two different electrode and microchannel designs, i.e., a “large” and a “small” biosensor design (see Figure 2).

*Immersion mode (IM).* An IM biosensor was used explicitly to optimize crucial steps in the development of the sensor, such as evaluating the cleaning quality of thin-film Au electrodes, establishing an appropriate method of mixed SAM deposition with the degree of enzyme immobilization on Au WE and for the characterization of the biosensor storage stability. IM refers to the biosensor system in which WE was the constructed thin-film electrode (Figure 1), placed at the bottom of the reservoir, while CE and RE were externally immersed into the electrochemical cell. CE was a Pt wire placed next to a miniature commercial Ag/AgCl RE with a liquid junction. For stability, both were attached to the housing via a proper mechanical support. The volume of the reservoir was 5 mL. It should be noted that a different enzyme immobilization strategy from that presented in Figure 1 was employed in conjunction with the IM biosensor. Here, a mixed SAM was activated by immersing the electrode chip with the thin-film electrodes separately into the solution of EDC and S-NHS for two hours, followed by immobilization of PQQ-GdhB. The enzyme was introduced by immersing the entire electrode chip into the enzyme solution for one day.

*Microfluidic mode (MM).* In addition to static (off-line) measurements, MM enables measurements during continuous flow chemistry, appropriate for, e.g., monitoring pharmaceutical processes using in-line, on-line or at-line systems. MM refers to all three thin-film electrodes being integrated into the electrode chip and encapsulated within the PDMS microchannel. Microfluidic biosensor construction is presented in detail in Figure 1. The flow of the solution through the channel was provided by a syringe pump with a flow rate between 0 and 100 µL/min.

*Large and small biosensor design*. The biosensor electrodes were of two different designs, large and small electrode design, as shown in Figure 2. The diameters of the circular WE were 3.0 and 0.8 mm for the large and small designs, respectively. Due to the different electrode designs, the microchannel width was adjusted to each of them. The microchannels had the same length and height, 20 mm and 0.1 mm, respectively, and two different widths, 6.7 mm and 1.2 mm, for the large and small sizes of electrodes, respectively.

### 2.4. Biosensor Glucose Detection Principle

**Figure 3:**
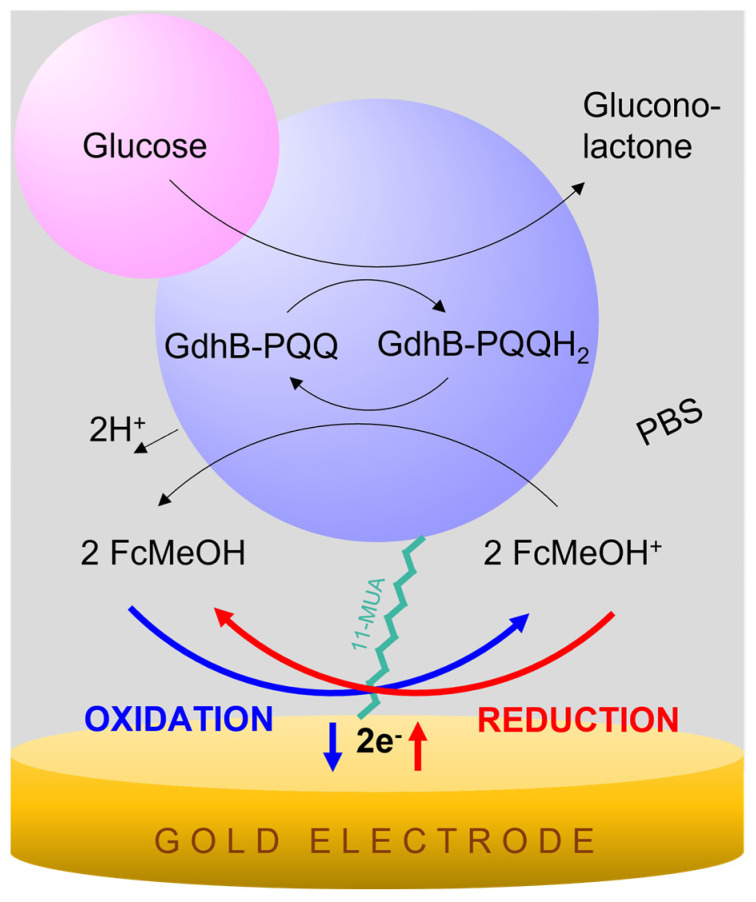
PQQ-GdhB biosensor reaction scheme for glucose detection in the presence of redox mediator FcMeOH^+^/FcMeOH in PBS electrolyte solution. Electrons pass indirectly from glucose and PQQ-GdhB to the electrode via mediator. Mediator oxidation is marked in blue and reduction in red.

The PQQ-GdhB sensor is based on the indirect transfer of electrons from the substrate–enzyme biochemical reaction to the electrode via the mediator redox couple FcMeOH^+^/FcMeOH, which is considered to diffuse freely in the PBS electrolyte solution. The reaction scheme applied to this type of biosensor is presented in Figure 3.

The substrate molecule glucose is oxidized in the presence of GdhB-PQQ producing gluconolactone and reduced GdhB-PQQH_2_, with the latter being further oxidized in the presence of reducing diffusional electron mediator. The mediator is reoxidized at a sufficiently positive potential, producing a current signal proportional to the glucose concentration—this represents chronoamperometric detection, marked in blue in Figure 3. In cyclic voltammetry, in addition to the oxidation process of the mediator, the reduction in the mediator is also detected, which is correspondingly marked in red.

## 3. Results and Discussion

In the first subsection, the results of the electrochemical measurements of the IM are presented, while in the second subsection the results of the MM are shown and discussed.

### 3.1. Immersion Mode Biosensor Characterization

The results presented for the IM biosensor refer to an experimental setup in which three electrodes (WE, CE, RE) were immersed in a reservoir containing the solution, as shown in Figure 2a. The results were obtained using a large and a small electrode.

**Figure 4:**
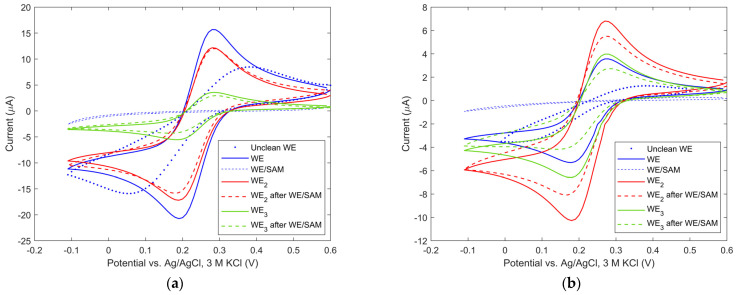
Cyclic voltammograms of 2 mM [Fe(CN)_6_]^3−/4−^ in an uncleaned, a cleaned and a WE/SAM-modified electrode chip with (**a**) large and (**b**) small electrode design in 0.1 M KCl supporting electrolyte.

*Evaluation of Au working electrode cleaning efficacy*. After electrode fabrication, the remaining contaminants must be removed from the surface of the electrode chip. The method of electrode chip cleaning by isopropyl alcohol, deionized water and oxygen plasma was described in Section 2.2. The cleanliness of the electrodes was investigated by cyclic voltammetry in the presence of [Fe(CN)6]^3−/4−^ in KCl solution by evaluating electrochemical reversibility. Figure 4a (dotted and solid blue curves) shows cyclic voltammograms obtained prior to and after electrode cleaning in oxygen plasma. The cyclic voltammogram was measured at a scan rate of 50 mV/s with 10 mV steps. As shown in Figure 4a, a difference in the cathodic and anodic peak current of 320 mV was measured prior to cleaning and 90 mV after cleaning. Therefore, the latter value is closer to the theoretical value of 57 mV for a single electron transfer reaction, which is characteristic of a pristine gold electrode surface [44]. In practice, peak voltage separation also depends on the resistance of the system and therefore differs from the theoretical one. In addition to plasma cleaning, we also obtained similar peak voltage separation within experimental uncertainty (91 mV with a standard deviation of 4 mV) using Piranha cleaning. Since both methods are considered rigorous in cleaning organic residues, the obtained values were considered to be a sufficient level of WE cleanliness. In addition, the process reversibility was tested by varying the scan rates from 20 mV/s to 120 mV/s. The reduction and oxidation peaks of the cyclic voltammograms followed a square root dependence of the scan rate, and the ratio of the anodic to cathodic peak current was ~0.98, which is close to 1, which is expected for a reversible process. This additionally confirms the proper cleanliness of our Au electrode.

*Evaluation of selectively mixed SAM deposition on the WE*. One of the challenges during the fabrication of the microfluidic biosensor was to deposit the mixed SAM only on the thin-film WE and not on the thin-film CE and RE, i.e., selective deposition on the WE. Mixed SAM deposition was performed by microcontact printing as described in Step 2 of Section 2.2. To evaluate the selectivity, we used three thin gold film electrodes correspondingly denoted as WE_1_, WE_2_ and WE_3_. Here each of the WEs was individually connected to the potentiostat, while CE and RE were external (IM, Figure 2a). Cyclic voltammetry was conducted before and after selective deposition of mixed SAM on each thin-film WE in the presence of ferri/ferro-cyanide in a KCl solution. The results obtained are reported by the three solid and three dashed traces in Figure 4a. Before deposition, each (oxidation and reduction) peak current was proportional to the surface area of the individual electrode (shown with solid traces). After depositing mixed SAM on the WE, the corresponding signal greatly decreased by 99% (shown with dashed blue trace) compared to that obtained using an unmodified WE. The formation of the mixed SAM on the Au electrode resulted in a highly insulating surface and thus blocked the Faradaic current. The anodic peak from the WE_2_ and WE_3_ after mixed SAM deposition on the WE remained almost the same (decreased by 2% and 17%, respectively; shown with dashed red and green traces), confirming the absence of mixed SAM contamination. This is solid proof that the deposition on the WE was specifically located, and no cross-contamination occurred on the other two thin-film electrodes WE_2_ and WE_3_. Figure 4b shows the results from the corresponding experiment involving a small electrode. After mixed SAM deposition on the WE, the signal from the mixed SAM-coated WE decreased by 99% (shown with dashed blue trace), compared to the signal from uncoated WE. The anodic peak from the WE_2_ and WE_3_ after mixed SAM deposition on the WE decreased by 19% and 31%, respectively (shown with dashed red and green traces). Despite the miniaturized electrode design, our deposition method proved to be suitable and still allowed selective deposition of mixed SAM without interference on the neighboring electrodes. As shown in the literature [45], the well-defined redox peaks commonly displayed on bare Au are drastically reduced for MUA-modified electrodes since the measured currents are at least one order of magnitude lower after the electrode functionalization. On the other hand, the FcMeOH redox couple in PBS does not show this isolating character for electron transfer through the mixed SAM to WE (as we obtain in the case of ferri/ferro-cyanide in KCl solution) and was selected for biosensor configuration.

**Figure 5:**
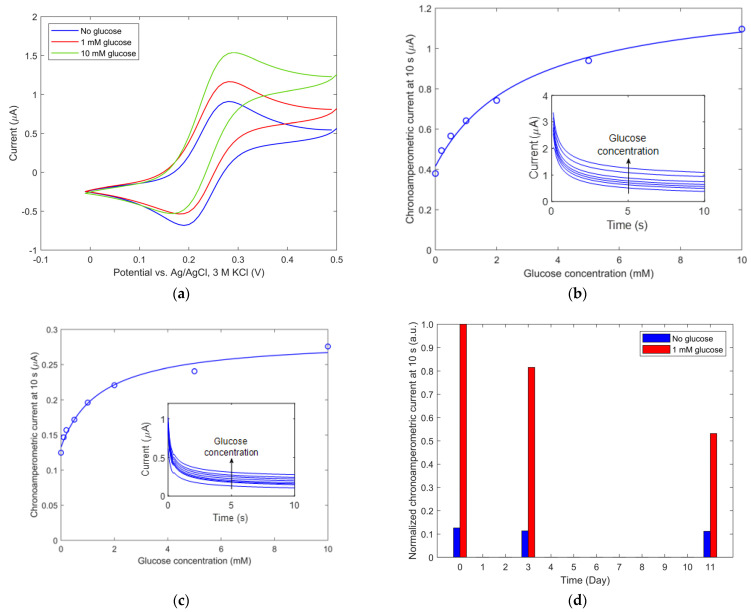
Immersion mode PQQ-GdhB biosensor electrochemical characterization: (**a**) Cyclic voltammogram for different glucose concentrations, measured with large electrodes; (**b**,**c**) chronoamperometric current dependency on glucose concentration, measured with large and small electrodes. Circles depict measured results and lines depict fitted results; (**d**) normalized chronoamperometric current dependence on storage time for 0 and 1 mM glucose.

*Glucose detection by IM biosensor.* The method of enzyme immobilization for the IM biosensor is described in Section 2.3. The efficacy of selective mixed SAM deposition together with enzyme immobilization on WE was analyzed by the response of the biosensor to glucose in the presence of FcMeOH in PBS. Figure 5a shows the cyclic voltammogram of the sensor response for different concentrations of glucose. The cyclic voltammogram is symmetrical when no glucose is present. As a response to the presence of glucose, a 27% (for 1 mM glucose) and 69% (for 10 mM glucose) increase in the anodic peak and a 22% decrease in the cathodic peak were observed, compared to the measurements in the absence of glucose. In the case without glucose, the ratio between FcMeOH^+^ and FcMeOH was equal, resulting in a symmetrical voltammogram, while in the case of glucose presence, the ratio was different due to additional electrons brought by the oxidation of glucose (see Figure 3), leading to an asymmetrical voltammogram. Chronoamperometric current response versus time was measured for glucose concentrations between 0.01 and 10 mM and was carried out at a constant DC potentiostatic setpoint potential E_DC_ of 0.35 V. Measured current values at 10 s are shown in a calibration plot (Figure 5b,c circles), while a full-time chronoamperometric response is shown in insets of Figure 5b,c. The response is in good agreement with the Cottrell equation [46] for simple redox events and diffusion-limited current dependence. The chronoamperometric current at 10 s in the range of 0.38 µA and 1.10 µA was measured in the case of large electrodes—see Figure 5b—and in the range of 0.12 µA and 0.28 µA in the case with small electrodes—see Figure 5c. The relation between measured current I and glucose concentration [*glucose*] is in good agreement with Michaelis–Menten kinetics and is confirmed with a fitted curve—see Figure 5b,c. Its formula is $I=I_{max}\frac{\left[glucose\right]}{K_{M}+\left[glucose\right]}+b$, where *I_max_* is the maximum current achieved by the system, *K_M_* is the Michaelis constant and *b* is the background current at zero glucose. The obtained parameters are *I_max_* = 0.86 µA, *K_M_* = 2.97 mM and *b* = 0.42 µA for large electrodes and *I_max_* = 0.15 µA, *K_M_* = 1.48 mM and b = 0.13 µA for small electrodes. The obtained Michaelis constants are in a good agreement with the data from the literature [36].

The stability of the developed biosensor was evaluated by measuring the chronoamperometric response signal at different storage time points: immediately, and then 3 and 11 days after biosensor construction. Between measurements, the biosensor was stored in PBS at 4 °C. Figure 5d shows the biosensor response normalized by an initial signal for 1 mM glucose. While the reference signal (measurement without glucose) remained constant, the signal for 1 mM glucose decreased with time. After 11 days, the decrease in current was about 45%. It is most likely that the decrease in signal is due to the loss of the PQQ-GdhB enzyme or its activity.

### 3.2. Microfluidic Mode Biosensor Characterization

All results concerning the MM biosensor refer to an experimental setup in which three electrodes (WE, CE, RE) are integrated into the microchannel enabling static or continuous flow (dynamic) measurements (see Figure 2b). The results refer to the large and small electrode design with appropriately adapted microchannels.

**Figure 6:**
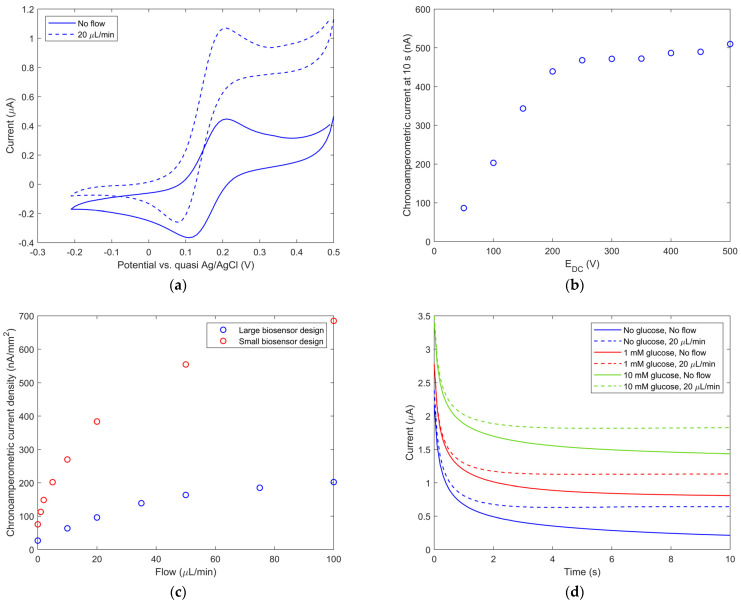

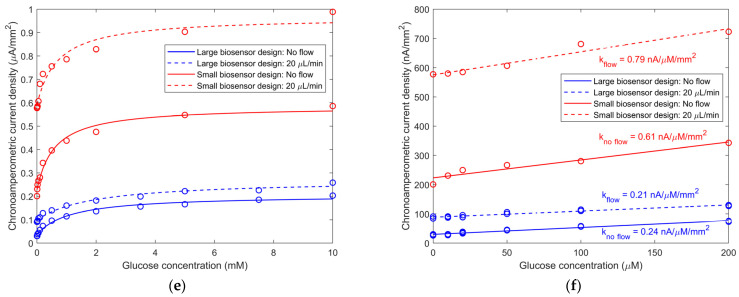
Characterization of microfluidic mode PQQ-GdhB biosensor: (**a**) Cyclic voltammogram obtained for zero glucose and with or without analytic solution flow; (**b**) chronoamperometric current vs. E_DC_ potential; (**c**) chronoamperometric current density values vs. flow rate for large and small electrode design without glucose; (**d**) time-dependent chronoamperometric current of large electrode design for various glucose concentrations and for 0 and 20 µL/min flow rates; (**e**) biosensor response for large and small electrode design for 0 and 20 μL/min flow rates, presenting measured values (circles) and Michaelis–Menten fitting plots (line); (**f**) linear range of biosensor response with calculated sensitivities k for glucose biosensors, presenting measured values (circles) and linear fitting plots (line).

*Potential and flow dependence.* MM characterization of the PQQ-GdhB biosensor on glucose is shown in Figure 6. The appropriate DC potential (E_DC_) for chronoamperometric measurements was determined from the cyclic voltammogram measured under static and dynamic conditions without glucose (see Figure 6a). The signal measured without flow is a typical symmetric peak-shaped reversible cyclic voltammogram, while the signal measured with flow has a 138% higher oxidation current due to the increased mass transport of reaction species to the electrode. Accurate potential E_DC_ was further determined and confirmed using chronoamperometry signals measured from 50 mV to 500 mV with a 50 mV step (see Figure 6b). From this dependence, an E_DC_ working potential of 0.25 V was chosen as the lowest possible potential that enables the highest response with minimal effects on other characteristics. This value is approximately 80 mV lower in comparison to the IM biosensor with external RE (see Figure 5a). This shift is a consequence of the potential difference between the miniature liquid junction Ag/AgCl in 3 M KCl and the thin-film quasi-Ag/AgCl RE, which is placed directly in the solution. The results of the chronoamperometric current density for the MM of large and small electrode designs as a function of flow rate are shown in Figure 6c. Considering the large electrodes, a linear dependence on flow rate is observed up to 40 μL/min, after which saturation occurs. On the other hand, the small electrodes exhibit the end of the linear regime below 5 μL/min. However, the chronoamperometric current density significantly increases even at higher flow rates. Due to sufficient sensitivity and repeatability, a flow rate of 20 μL/min was chosen for further measurements at the large and the small electrodes.

*Glucose detection by MM biosensor.* Glucose solutions with concentrations from 10 μM to 10 mM were injected by a syringe pump into the microchannel of the microfluidic biosensor one at a time and detected by a chronoamperometric technique. The results for three selected cases of glucose concentration (0 mM, 1 mM, 10 mM) are shown in Figure 6d. The proportional increase in the electric current with increasing glucose concentration and flow was observed. The biosensor signal decreases with time when measured without flow, which is consistent with Cottrell’s equation, while in the presence of flow, after the initial few seconds, the biosensor signal remains independent of time. The transport of glucose to the activation sites of the enzyme is, in the case of zero flow, limited by diffusion, while in the case of flow, the convection transport mechanism ensures continuous access of the glucose to the reaction sites of the enzyme.

The current density sampled after 10 s of measurement is presented in Figure 6e. In the case of the large electrode design, the oxidation current density increases from 30 nA/mm^2^ to 203 nA /mm^2^ without flow and from 91 nA/mm^2^ to 259 nA/mm^2^ with flow. In the case of the small electrode design, an increase in oxidation current density from 201 nA/mm^2^ to 586 nA/mm^2^ and from 577 nA/mm^2^ to 988 nA/mm^2^ was observed without flow and with a flow of 20 µL/min, respectively. The results in Figure 6e also confirm that the biosensor response follows standard Michaelis–Menten enzyme kinetics. Therefore, the main three advantages of biosensors performing in the microfluidic channel are as follows: (i) continuous detection of the analyte is enabled; (ii) the flow through biosensor structure gives a higher response; and (iii) measurement readings are time-independent in the case of flow. Based on the calibration plot in Figure 6e, the upper limit of the linear range for both biosensor designs was at a glucose concentration of 200 µM.

Comparing the large and small electrodes, a pronounced increase in current density is shown by using the small electrode design. Regardless of the glucose concentration, about 3× higher current density is observed (Figure 6e). Moreover, an improvement is also shown when comparing biosensor sensitivities *k* (see Figure 6f). Here, based on the slopes of the respective calibration plots, a sensitivity of 0.21 and 0.24 nA/μM/mm^2^ was estimated at large electrodes and 0.79 and 0.61 nA/μM/mm^2^ at small electrodes, with and without flow, respectively. To summarize, approximately, a factor of 3 in sensitivity improvement is achieved when moving from the large electrode design to the small biosensor design in MM with continuous analyte flow.

The chronoamperometric response of the developed microfluidic PQQ-GdhB glucose biosensor at different glucose concentrations was further analyzed to determine the limit of detection (LOD). The LOD was calculated according to the formula 3* std/*k* [30], where std is the standard deviation of the chronoamperometric current of the blank sample (without glucose) and sensitivity *k* is the slope in the linear range of the calibration plot (0–200 μM) (Figure 6f). According to the methodology described above, the biosensor LOD was determined to be 30 μM.

The materials used for fabrication of the biosensor, and the sensitivity, linear range and LOD have been compared with the existing glucose electrochemical biosensors (Table 1).

Comparing the analytical performances of the proposed biosensor with various glucose electrochemical biosensors shows that the thin-film Au PQQ-GdhB sensor had a relatively highly sensitive response to glucose but only in the quite limited linear range of 30–200 μM. However, our biosensor still enables measurements up to 10 mM with reduced and nonlinear sensitivity. Additionally, we use mature microelectromechanical system technology, which makes the production more accessible.

## 4. Conclusions

We presented the development and characterization of an electrochemical biosensor in a microfluidic system for glucose measurement. Each step of the biosensor construction was verified separately, using IM and MM approaches. An IM biosensor was used for static measurements to optimize crucial steps regarding enzyme immobilization on the gold WE, while the MM biosensor was used for measurements in both static and continuous flow systems. The enzyme PQQ-GdhB was immobilized within the microchannel on the WE, where a mixed SAM of 6-MCH and 11-MUA was precisely deposited via microcontact printing and activated by crosslinker EDC+S-NHS. The advantage of microcontact printing for efficient mixed SAM deposition is a much shorter process, which was typically a few minutes in our work, compared to several hours for conventional deposition. Another advantage of this approach is that the complete fabrication of the device can be performed in advance, while the enzyme with the highest activity is applied to glucose detection. Two different sizes of thin film electrodes were used—large (WE diameter is 3.0 mm) and small (WE diameter is 0.8 mm) electrodes—both with customized microchannel designs. It has been shown that a fabricated microfluidic biosensor can detect a wide range of glucose concentrations from 30 µM to 10 mM. The response of the biosensor showed that the biosensor with the small electrode and microchannel design outperformed the biosensor with the large electrode and microchannel design in terms of current density. The sensitivity of the biosensor without flow is 0.61 nA/µM/mm^2^ in the linear range of the calibration plot, which is between 30 and 200 µM in glucose concentration. In the presence of a continuous glucose flow of 20 µL/min, the sensitivity of the biosensor further increased to 0.79 nA/µM/mm^2^. Good sensor sensitivity in this study under flow shows that the constructed biosensor is suitable for monitoring pharmaceutical processes. The benefit of our biosensor development approach is in the fabrication process, which comprises mature microelectromechanical system technology and enables a large-scale biosensor fabrication with high repeatability. While the biosensor was designed primarily for monitoring glucose in pharmaceutical processes, it also has potential for point-of-care testing. However, for those applications, validation of the sensor selectivity in biological samples would have to be performed. The presented development of a biosensor on a chip can be extended with automatization, various microfluidic components and multiplexed continuous biosensing.

## Figures and Tables

**Table 1:** Performance comparison of proposed biosensor with existing glucose electrochemical biosensors.

Materials	Sensitivity (μA mM^−1^ cm^−2^)	Linear Range (mM)	LOD (μM)	Ref.
ZnO nanosheets microspheres	210.8	0.05–23	50	[47]
CoWO4/CNT	10.89	0.05–0.3	1.3	[48]
Prussian blue enzyme	70.76	0.05–3.15	10	[49]
Porous graphene	65.6	0.0003–2.1	0.3	[50]
Fluorocarbon	114	0.03–1.1	15	[51]
Three-dimensional hydrogen titanate nanotubes	1.541	1–10	59	[52]
Au-multiwalled carbon nanotubes	405.2	0.05–5	3	[53]
Graphene quantum dots	21.64	0–0.5	65	[54]
A biocompatible conjugated polymer	12.69	1–30	4.7	[55]
Vertically aligned carbon nanotube	1462	1.2–7.8	23	[56]
Nafion solubilized Au/MXene nanocomposite	4.2	0.1–18	5.9	[57]
Functional GOx/silica–lignin system	0.78	0.5–9	145	[58]
Thin film Au PQQ-GdhB	79	0.03–0.2	30	This work

## Data Availability

Data are available on request from the authors.
